# The East Tennessee, Virginia & Georgia Tariff

**Published:** 1892-01

**Authors:** 


### THE EAST TENNESSEE, VA. & GA. TARIFF.

The November issue of the RECORD contained an editorial upon the subject of the tariff charges that have been adopted by many railroads, and in comparing them found it necessary to criticise especially that of the East Tennessee, Virginia and Georgia, as being much lower than any other of the roads doing business in Atlanta.

A communication has been received from Dr. W. C. Jarnagin, the representative of this company in the city, in which he claims that an injustice has been done him by the article in question. He states that before accepting the position the tariff was modified by the Chief Surgeon, and that he is not called to comply strictly with its demands, which of course frees him from anything personal in the criticism, as it was only directed against the official tariff promulgated by the railroad.

We take pleasure in publishing the statement of Dr. Jarnagin, and in assuring him that the article was not of a personal nature, but we do not wish to be understood as in any way withdrawing anything that applies to the company's schedule as placed upon the surgeons at other points.

